# Electronic Heart (ECG) Monitoring at Birth and Newborn Resuscitation

**DOI:** 10.3390/children11060685

**Published:** 2024-06-04

**Authors:** Sarah Mende, Syed Ahmed, Lise DeShea, Edgardo Szyld, Birju A. Shah

**Affiliations:** 1 Department of Pediatrics, College of Medicine, University of Oklahoma (OU), Oklahoma City, OK 73104, USA; 2 Department of Pediatrics, University of Texas Southwestern Medical Center, Dallas, TX 75390, USA; 3 Neonatal-Perinatal Medicine, Department of Pediatrics, Indiana University School of Medicine, Indianapolis, IN 46202, USA; 4 Neonatal-Perinatal Medicine, Oklahoma Children’s Hospital at OU Health, 1200 North Everett Drive, 7th Floor North Pavilion ETNP #7504, Oklahoma City, OK 73104, USA

**Keywords:** electrocardiogram, assessment, heart rate, measurement, neonate, infant, resuscitation, delivery, team performance, outcomes

## Abstract

Background: Approximately 10% of newborns require assistance at delivery, and heart rate (HR) is the primary vital sign providers use to guide resuscitation methods. In 2016, the American Heart Association (AHA) suggested electrocardiogram in the delivery room (DR-ECG) to measure heart rate during resuscitation. This study aimed to compare the frequency of resuscitation methods used before and after implementation of the AHA recommendations. Methods: This longitudinal retrospective cohort study compared a pre-implementation (2015) cohort with two post-implementation cohorts (2017, 2021) at our Level IV neonatal intensive care unit. Results: An initial increase in chest compressions at birth associated with the introduction of DR-ECG monitoring was mitigated by focused educational interventions on effective ventilation. Implementation was accompanied by no changes in neonatal mortality. Conclusions: Investigation of neonatal outcomes during the ongoing incorporation of DR-ECG may help our understanding of human and system factors, identify ways to optimize resuscitation team performance, and assess the impact of targeted training initiatives on clinical outcomes.

## 1. Introduction

Transitioning from intrauterine to extrauterine life involves multiple cardiac and pulmonary structural and physiologic changes. The fetus must adapt quickly from a primarily hypoxemic environment and transition from placental oxygenation to oxygenation via the lungs at the time of delivery [1]. Labor and initiation of the neonate’s first breath trigger many of these crucial changes, including a dramatic decrease in pulmonary vascular resistance, increase in systemic vascular resistance, reversal of flow across the ductus arteriosus, closure of fetal shunts, increase in heart rate (HR), clearance of lung fluid, and establishment of breathing patterns [1,2,3]. Successful gas exchange is typically established by 2 min of life, at which point the HR should also increase to >100 bpm. During this crucial transition, changes in heart rate are the most important vital sign indicating appropriate ventilation [1,4].

In the first minutes of life, resuscitation is focused on ventilation. The crucial physiologic transitions mentioned above are highly dependent on the lungs taking over ventilation. It stands to reason, then, that the primary need for resuscitation in the neonatal period is due to abnormalities hindering successful ventilation [5]. Lack of proper transition described above leads to the need for resuscitation in approximately 10% of neonates. Of this 10%, approximately 1% of neonates will require more advanced resuscitative efforts such as intubation and chest compressions. Resuscitative measures are dependent on many clinical factors. The neonatal resuscitation team considers HR and oxygen saturation. Among these factors, HR is the main determining vital sign for the next steps in resuscitation based on the guidelines provided by the American Heart Association (AHA) and American Association of Pediatrics (AAP) [5,6,7].

Oxygen and ventilation support are administered based on clinical assessment and whether HR is <100 bpm. Because HR is the most important clinical indication of successful ventilation, it is vital that the method of measuring HR in the delivery room (DR) is accurate and provides results in a timely manner [8,9]. Multiple known and novel methods can be used to measure HR in the DR: auscultation, palpation, pulse oximetry (PO), and electrocardiogram (DR-ECG). Traditionally, auscultation and palpation have been the standard, but they typically underestimate HR for HR >100 bpm. Neither of these methods provide continuous monitoring unless it is the sole role of a staff member, which is often unreasonable with limited staffing and can be difficult to maintain during advanced resuscitation [7,9,10]. Unlike palpation and auscultation, PO allows for continuous monitoring of HR, but PO typically underestimates HR before 2 min of life due to poor perfusion of the extremities. In contrast, DR-ECG provides continuous, accurate, and early measurement of HR [6,9,10]. Multiple studies have shown that DR-ECG provides accurate HR parameters faster than other methods [8,11]. In 2016, the American Heart Association updated its guidelines to suggest the use of electrocardiographic (ECG) leads for accurate neonatal heart rate monitoring in infants receiving resuscitation [6].

Our Level IV neonatal intensive care unit (NICU) at Oklahoma Children’s Hospital integrated routine use of three-lead ECG in the DR for neonates receiving positive pressure ventilation (PPV) or higher support in 2017. Previous investigations by our team evaluated patterns of DR interventions between pre-implementation (2015) and post-implementation (2017) cohorts of ECG use in the DR [12]. The objectives of this study were to evaluate serial trends in the frequency of DR interventions, comparing results from a recent cohort of infants born in 2021 with those of infants born in 2015 and 2017.

## 2. Methods

This longitudinal cohort study analyzed maternal and infant data abstracted from medical records by trained staff at Oklahoma Children’s Hospital at the University of Oklahoma Health Sciences Center. Participants included in-born infants admitted to our hospital who received PPV or higher support in the DR. This study included cohorts from 2015 (pre-implementation of ECG use), 2017 (upon implementation), and 2021 (4 years post-implementation).

The cohorts were compared on maternal demographics, perinatal factors, DR interventions, and neonatal outcomes. Maternal demographics were extensive including variables such as age, primigravida, receipt of prenatal care, substance use (tobacco, alcohol, or drugs), maternal diagnoses such as diabetes mellitus and pre-eclampsia, and treatments received during labor such as steroids and/or magnesium. Perinatal variables included multiple gestation, fetal growth restriction, fever, group-B streptococcus positive, chorioamnionitis, rupture of membranes > 24 h, antibiotics, antepartum hemorrhage, abnormal fetal heart rate pattern, meconium-stained fluid, cord accidents, nuchal cord, shoulder dystocia, vacuum delivery, emergency c-section, and anesthesia. DR variables included oxygen use, PPV, continuous positive airway pressure (CPAP), tracheal intubation, chest compressions, epinephrine use, and Apgar scores (1, 5 and 10 min after birth). We also investigated neonatal death during the hospital stay. Groups were compared using generalized linear models with the subjects nested in cohorts. Binary outcomes were analyzed using a logistic link function, and a Gaussian link was used for continuous outcomes.

## 3. Results

Table 1 provides median (IQR) and *n* (%) for maternal demographics, perinatal factors, and infant variables for the pre-implementation (2015) and post-implementation (2017, 2021) cohorts. Table 2 compares the cohorts on DR variables and interventions as well as in-hospital neonatal mortality. Positive pressure ventilation use at birth was significantly higher in the post-implementation cohorts (2017, 2021) compared to pre-implementation (2015). Cohort 2017′s higher chest compressions compared to 2015 showed a trend toward significance (*p* < 0.10), then the rate decreased significantly from 2017 to 2021, when the rate was statistically indistinguishable from 2015. Tracheal intubations decreased from 2015 to 2017, then increased in 2021, returning to a rate that was statistically equivalent to that of 2015.

Table 2 also shows tracheal intubation rates separated by gestational age (<34 weeks and ≥34 weeks). For infants born at less than 34 weeks, there was a significant increase in the frequency of tracheal intubation from 2017 to 2021. Among infants born at 34 weeks or higher gestational ages, the frequency of tracheal intubation was significantly lower in 2017 and 2021 compared with 2015.

Further analyses and documentation of other maternal, perinatal characteristics, and neonatal outcomes are available for review in the Appendix A.

## 4. Discussion

After sustained implementation of ECG for heart rate evaluation in the DR, the 2021 cohort showed a decrease in the use of chest compressions and an increase in the frequency of tracheal intubations compared to the initial post-implementation group in the 2017 cohort. When comparing the 2021 cohort with the pre-implementation cohort (2015), there was no significant difference in the usage of tracheal intubation or chest compressions. These findings differ from the previous study from our research program [12]. In our original study, there was a slight but non-significant increase in chest compressions and a significant decrease in tracheal intubations in the 2017 cohort compared to the 2015 cohort. Despite these differences between both post-implementation groups, there were no significant differences in mortality among the cohorts.

Multiple factors could explain the significant differences between the use of DR chest compressions and tracheal intubation among post-implementation cohorts. Possible contributing factors include human factors and variability, especially with a change in procedure on a facility-wide scale. The Neonatal Resuscitation Program (NRP^®^) textbook outlines a straightforward algorithm for healthcare providers, but as with any complex care setting, an influx of multiple stimuli taxes people’s ability to perform optimally. In an effort to counter this human element, a system-wide interdisciplinary project was launched in 2019, targeting NRP providers and instructors on the timely implementation of establishing early rescue airway to effectively deliver positive pressure ventilation during troubled transitions at birth [13]. Focused education interventions on the importance of establishing a reliable method of ventilation likely contributed to the decreased frequency of chest compressions and increased frequency of intubations in the 2021 cohort compared to the 2017 cohort.

When tracheal intubation rates were examined based on gestational age, the frequency of tracheal intubation among preterm infants (<34 weeks) in the 2021 cohort was comparable to tracheal intubation rates described by previous studies from facilities of a similar size. A study investigating the efficacy of quality improvement interventions in the DR for infants born between 22–29 weeks gestational age showed tracheal intubation rates of 50–58% [14]. These rates subsequently decreased following quality improvement measures in the DR. In another quality improvement study for decreasing tracheal intubation rates for preterm infants (22–29 weeks), initial tracheal intubation rates among three different groups ranged from 43–53% [15]. Although we observed an increase in tracheal intubation rates, the most recent findings from 2021 are consistent with pre-term tracheal intubation rates described by other studies. For infants born at 34 or greater weeks gestational age, previous studies also showed similar findings prior to initiating measures for improving quality [16]. These results highlight the potential to refine practices at our facility.

Between 2017 and 2021 our facility underwent multiple changes that may have also contributed to the differences seen between these two post-implementation groups. As another method for quality improvement in the DR, our facility incorporated dedicated transition nurses in labor and DR. These systematic measures provide a possible explanation for our findings over recent years since routine DR-ECG use began. COVID-19 and the institutional challenges associated with the pandemic may have also affected these findings. Other factors that could have affected the frequency of different resuscitation methods were strain on already limited staffing, finite organizational resources as well as variable levels of training, and awareness and experience among frontline providers over the course of the COVID-19 pandemic.

The limitations of this study include the retrospective nature of data collection as well as limited data on long-term neonatal outcomes. This study could not be randomized, leaving potential confounders uncontrolled, and causality cannot be deduced from our findings. Although long-term outcomes were beyond the scope of this study, they are an important area for further investigation. Among the strengths of this study were the inclusion of extensive maternal and perinatal variables and the robust sample size.

## 5. Conclusions

We have demonstrated that ECG implementation in the DR can be sustained at a large, academic, tertiary care Level IV NICU despite COVID-19 pandemic and other organizational challenges. DR-ECG may advance our understanding of human and systemic factors, including effects on the resuscitation team, when HR information is available at birth in real time. Therefore, further incorporation of electronic heart monitoring during neonatal resuscitation needs systematic evaluation to illuminate its impact on the delivery room interventions, clinical outcomes, team performance, human factors, and hospital resources.

## Figures and Tables

**Table 1:** Demographics of the study population.

Characteristic	2015, *n* = 263 *n* (%) or Median (IQR)	2017, *n* = 369 *n* (%) or Median (IQR)	2021, *n* = 379 *n* (%) or Median (IQR)
Maternal			
Age, years	27 (22–32)	27 (23–31)	28 (24, 33)
Primigravida	70 (26.7)	111 (30.1)	109 (28.8)
No prenatal care	9 (3.4)	18 (4.9)	24 (6.3)
Tobacco, alcohol, illicit drug use	41 (15.6)	82 (22.2)	83 (21.9)
Diabetes mellitus	24 (9.1)	45 (12.2)	47 (12.4)
Pre-eclampsia	79 (30.0)	140 (37.9)	135 (35.6)
Steroids	128 (48.7)	236 (64.0)	223 (58.8)
Magnesium	82 (31.2)	177 (48.0)	144 (38.0)
Perinatal			
Multiple gestation	38 (14.4)	58 (15.7)	52 (13.7)
Fetal growth restriction	22 (8.4)	23 (6.2)	46 (12.1)
Fever	10 (3.8)	8 (2.2)	12 (3.2)
Group-B streptococcus			
Positive	46 (17.5)	73 (19.8)	70 (18.5)
Unknown	93 (35.4)	141 (38.3)	143 (37.7)
Chorioamnionitis	26 (9.9)	28 (7.6)	20 (5.3)
Rupture of membranes, >24 h	30 (11.4)	32 (8.7)	55 (14.5)
Antibiotics	59 (22.4)	109 (29.5)	90 (23.7)
Antepartum hemorrhage	13 (4.9)	9 (2.4)	23 (6.1)
Abnormal fetal heart rate pattern	57 (21.7)	80 (21.7)	97 (25.6)
Meconium-stained amniotic fluid	31 (11.8)	35 (9.5)	46 (12.1)
Cord accidents	4 (1.5)	17 (4.6)	11 (2.9)
Nuchal cord	25 (9.5)	26 (7.0)	32 (8.4)
Shoulder dystocia	1 (0.4)	6 (1.6)	17 (4.5)
Forceps or vacuum delivery	6 (2.3)	13 (3.5)	5 (1.3)
Cesarean delivery	193 (73.4)	270 (73.2)	289 (76.3)
Emergency	138/193 (71.5)	202/270 (74.8)	173/289 (59.9)
Anesthesia	237 (90.1)	318 (86.2)	357 (94.2)
General	44/237 (18.6)	62/318 (19.5)	58/357 (16.2)
Cord pH	7.2 (7.17–7.29)	7.2 (7.14–7.31)	7.3 (7.19, 7.30)
Cord pCO_2_	56.5 (46.8–65.8)	55.0 (46.0, 67.5)	56.0 (49.0, 64.0)
Cord base deficit	5.5 (3.0–7.3)	5.0 (3.0–9.0)	4.0 (2.0–6.0)
Infant			
Gestation, weeks	33.9 (29.1–37.3)	33.6 (30.1–36.7)	34.0 (30.0–37.0)
Birth weight, grams	2080 (1230–2915)	2100 (1320–2750)	2070 (1321, 2900)
Male	135 (51.5)	191 (51.8)	187 (49.5)

IQR = interquartile range (25th, 75th percentiles).

**Table 2:** Delivery room variables and in-hospital mortality.

Variable	2015, *n* = 263 *n* (%) or Median (IQR)	2017, *n* = 369 *n* (%) or Median (IQR)	2021, *n* = 379 *n* (%) or Median (IQR)
Positive pressure ventilation	239 (91.9) ^A,B^	360 (97.6) ^A^	365 (96.3) ^B^
Tracheal intubation	125 (47.5) ^A^	131 (35.5) ^A,B^	166 (43.8) ^B^
Infants < 34 weeks	71/133 (53.4)	88/194 (45.4) ^A^	108/185 (58.4) ^A^
Infants ≥ 34 weeks	54/130 (41.5) ^A,B^	43/175 (24.6) ^A^	58/194 (29.9) ^B^
Chest compressions	8 (3.0)	24 (6.5) ^A^	8 (2.1) ^A^
Epinephrine use	1 (0.4)	5 (1.4)	7 (1.9)
Apgar scores			
1 min	3 (2, 6)	4 (2, 6)	4 (2, 6)
5 min	6 (5, 8)	7 (5, 8)	7 (5, 8)
10 min	7 (7, 8)	7 (7, 8)	7 (7, 8)
Supplemental oxygen	224 (85.2) ^A^	330 (89.4) ^B^	378 (99.7) ^A,B^
Continuous positive airway pressure	186 (70.7) ^A,B^	323 (87.5) ^A^	329 (86.8) ^B^
In-hospital mortality	23 (8.7)	30 (8.1)	32 (8.4)

Columns that share the same superscript on a row differed significantly, *p* < 0.05. IQR = interquartile range (25th, 75th percentiles).

## Data Availability

The de-identified data presented in this study are available on request from the corresponding author. The data are not publicly available due to compliance with HIPAA.

## Appendix A

Generalized linear models and a logistic linking function were run for all of the outcomes in Table 2, with the exception of Apgar scores for which a Gaussian linking function was used. Unlike the multivariable analyses reported in Shah et al. [12], this paper omitted the covariates of race and use of forceps or vacuum because of sparse cells making the analysis untenable. Otherwise, the same covariates were included in the models:

- Documented use of tobacco, alcohol, or illicit drugs
- Pre-eclampsia
- Diabetes mellitus
- Antenatal antibiotics
- Intrauterine growth restriction
- Steroids
- Magnesium
- Meconium
- Any cord accident
- Abnormal hearth pattern
- Antepartum hemorrhage
- General anesthesia
- Urgent Cesarean delivery
- Gestational age (weeks)

Table A1 shows the pattern of results for each outcome from the unadjusted analyses reported in Table 2 and the pattern of results from the adjusted (multivariable) analyses; an equal sign means the rates for that outcome did not differ significantly between the years shown, while a directional sign indicates a significant difference in the direction shown. Rows have been added under each outcome to list the significant covariates (*p* < 0.05). The last column shows those covariates’ odds ratios or, in the case of Apgar scores, regression coefficients, as well as their associated 95% confidence intervals.

**Table A1:** Multivariable analysis of delivery room variables and neonatal mortality.

Outcome Variable	Unadjusted Comparison of Years	Adjusted Comparison of Years	Odds Ratios (95% CI) or Regression Coefficient (95% CI)
Positive pressure ventilation	2015 < 2017 = 2021	2015 < 2017 = 2021	
Steroids			3.63 (1.46, 9.43)
Cord accident			0.24 (0.07, 0.98)
Gestational age			1.11 (1.02, 1.22)
Tracheal intubation	2017 < 2015 = 2021	2015 = 2021	
		2017 = 2021	
		2015 > 2017	
Steroids			0.41 (0.26, 0.65)
Meconium			1.97 (1.12, 3.49)
General anesthesia			2.01 (1.33, 3.03)
Urgent Cesarean			1.56 (1.03, 2.35)
Gestational age			0.81 (0.77, 0.85)
Chest compressions	2015 = 2021 > 2017	2015 = 2021 > 2017	
Abn. heart pattern			3.37 (1.48, 7.77)
General anesthesia			3.29 (1.45, 7.52)
Gestational age			0.86 (0.78, 0.95)
Epinephrine use ^a^	2015 = 2017 = 2021	2015 = 2017 = 2021	
APGAR score, 1 min	2015 = 2017 = 2021	2015 = 2017 = 2021	
Steroids			1.04 (0.59, 1.49)
Meconium			−0.69 (−1.29, −0.10)
Abn. heart pattern			−0.73 (−1.14, −0.32)
General anesthesia			−1.06 (−1.47, −0.64)
Gestational age			0.12 (0.08, 0.17)
APGAR score, 5 min	2015 = 2017 = 2021	2015 = 2017 = 2021	
Steroids			0.69 (0.30, 1.07)
Cord accident			0.74 (0.01, 1.48)
General anesthesia			−0.83 (−1.18, −0.47)
Gestational age			0.13 (0.08, 0.17)
APGAR score, 10 min	2015 = 2017 = 2021	2015 = 2017 = 2021	
Pre-eclampsia			0.41 (0.01, 0.80)
Steroids			0.47 (0.06, 0.88)
Meconium			−0.56 (−1.08, −0.04)
Gestational age			0.09 (0.05, 0.14)
Supplemental oxygen	2021 > 2015 = 2017	2021 > 2017 > 2015	
Cord accident			0.28 (0.09, 0.95)
Continuous positive airway pressure	2015 < 2017 = 2021	2015 < 2017 = 2021	
Antibiotics			0.57 (0.35, 0.93)
Steroids			3.59 (2.03, 6.49)
General anesthesia			0.60 (0.38, 0.98)
Gestational age			1.16 (1.10, 1.23)
Survival ^c^	2015 = 2017 = 2021	2015 = 2017 = 2021	
Pre-eclampsia			2.83 (1.34, 6.33)
IUGR ^b^			0.27 (0.14, 0.54)
Steroids			2.41 (1.17, 5.05)
Gestational age			1.20 (1.11, 1.30)

^a^ No significant covariates, all *p* > 0.05. ^b^ IUGR = Intrauterine growth restriction. ^c^ This variable was reported as in-hospital neonatal mortality in Table 2 to be consistent with Shah et al. [12], but in fact it was coded as survival (1 = yes, 0 = no). For interpretation of odds ratios for the covariates, the variable needs to be reported as it was coded.
